# Author Correction: Psychological measures aren’t toothbrushes

**DOI:** 10.1038/s44271-024-00086-5

**Published:** 2024-04-22

**Authors:** Malte Elson, Ian Hussey, Taym Alsalti, Ruben C. Arslan

**Affiliations:** 1 Institute of Psychology, University of Bern, Fabrikstrasse 8, 3012 Bern, Switzerland; 2 Department of Psychology, University of Leipzig, Leipzig, Germany

**Keywords:** Psychology, Human behaviour, Research data

Correction to: *Communications Psychology* 10.1038/s44271-023-00026-9, published online 17 October 2023

In the original version of Fig. 2, the lower panel contained a plot in which all measures of Shannon entropy were incorrectly normalized; normalization in that version used the log of the number of times each measure was used. The lower panel in the corrected Fig. 2 plots Shannon entropy normalized by the number of measures that were used at least once.
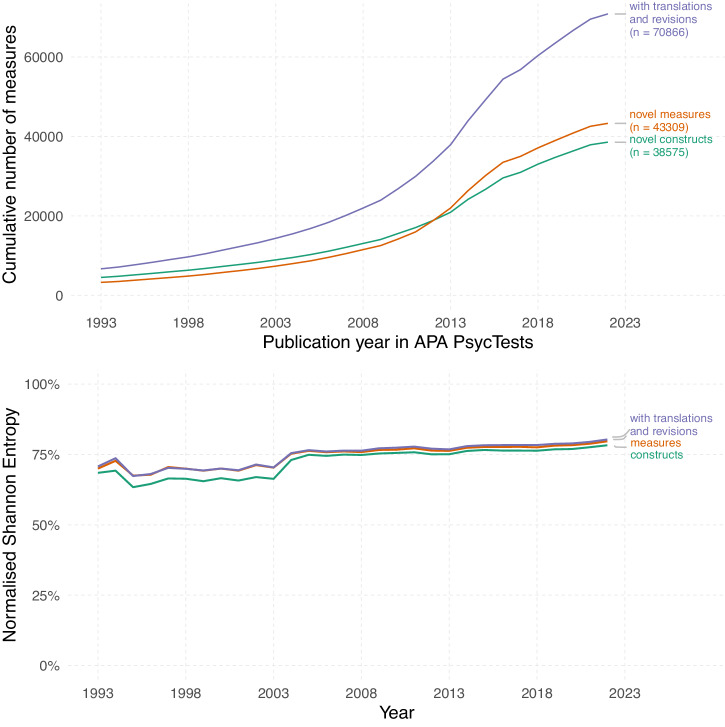


The caption for Fig. 2 has been corrected accordingly. The sentence: “The literature does not converge on standards over time, but rather is becoming increasingly fragmented.” Has been replaced with: “The literature does not converge on standards over time, but rather remains fairly consistently fragmented.”

The Supplementary Information file contained a typesetting error so that Equation number 1 appeared once in LaTex notation and once, additionally, typeset appropriately. The corrected Supplementary Information file does not contain the superfluous LaTex notation.

After correcting this mistake, we see a less pronounced increase in normalised entropy over time and overall higher levels of entropy (i.e. higher fragmentation).

### Supplementary information

Elson_SI_corrected

