# Supplementary material for: Psychological measures aren’t toothbrushes
Source: Commun Psychol. 2023 Oct 17;1:25. doi: 10.1038/s44271-023-00026-9 (PMC11332227; doi:10.1038/s44271-023-00026-9)
Supplement: Supplementary file 1 — Supplementary Information [file 44271_2023_26_MOESM1_ESM.pdf]

Psychological Measures Aren't Toothbrushes

Malte Elson
Ian Hussey
University of Bern

Taym Alsalti
Ruben C. Arslan
University of Leipzig

Supplementary Information (Figures 1 and 2)
The American Psychological Association (APA) maintains several databases: PsycTests records measures and instruments used in psychological research. APA staff continuously take this information from journal articles, technical reports, and dissertations, and authors of tests and measures are invited to submit them directly to APA. Each record provides a summary of the measure, the construct it supposedly operationalises, and bibliographic information (e.g., authors, publication year), as well as psychometric information on reliability, validity, and factor analyses when that data is reported in the source document. Revisions and translations of existing tests are also linked to their originals. Records include supplemental information such as datasets, answer keys, scoring sheets, and test manuals, when available. As of August 21, 2023, there are 73,646 records in PsycTests according to APA.

PsycInfo is a large database of research in the social and behavioural sciences with over 5,000,000 peer-reviewed records, according to the APA. Among many other things, records have a field for "test and measures" where the names of measures used in an empirical study are recorded. If a record of a measure also exists in PsycTests, it is often (though not consistently) cross-referenced in this field.

We used the PsycTests database to generate a master list of measures used in psychological research by looking up tests using their specific test DOI. This way, different measures with the same generic name (such as "job satisfaction scale") could be distinguished. We then counted the publications using each measure in each year from 1993 to 2022. For Figure 1, we simply aggregated counts across years and plotted how often measures tended to be used. However, 27,000 tests recorded in APA's PsycTests database (56% of those published between 1993 and 2022) have no matches recorded in the PsycInfo database at all. Therefore, Figure 1 is likely undercounting the number of times a measure has only been used once.

For Figure 2A, we generated cumulative counts of distinct measures and constructs. In one specification, we excluded revisions and translations from our count of new measures, in another we included it. For Figure 2B, we computed a measure of fragmentation for each year. We computed fragmentation of measures and constructs. Lumping translations and revisions with the original measure on which they were based made little difference to the results.

Our measure of fragmentation is normalised Shannon entropy, sometimes referred to as H_{rel} (Eq. 1; Wilcox, 1973). The maximal Shannon entropy of a set of tests rises with the number of tests that were used at least once `log(n)`. So as not to restate the result that the number of tests increases over time, we normalise entropy by `log(n)`. The maximum is therefore 1 and would be reached if, in each year, all tests are used an equal number of times across publications. The minimum is 0 and would be reached if, in a given year, all publications used the same measure.

Eq. 1: $$\eta(X) = -\sum_{i=1}^{n} \frac{p(x_i)\log_b(p(x_i))}{\log_b(n)}$$

REFERENCES

Wilcox, A. R. (1973). Indices of qualitative variation and political measurement. *Western Political Quarterly, 26*(2), 325–343.

Category	Things to look out for
Nonredundancy	- Are correlations with other measures reported?<br>- How were the other measures selected (e.g., based on face validity)?<br>- Was the correlation analysis conducted on the same sample used for the focal analyses or on a separate validation sample?<br>- Were correlation coefficients corrected for measurement error (e.g., through latent variable modelling)?<br>- Does the correlations analysis show that the new measure has incremental validity over conceptually similar measures?
Protocol adherence	- Do the authors report having adhered to a published measurement procedure?<br>- To what extent did the authors actually adhere to it (see measure modifications)?
Comprehensive Reporting	- Were all elements of the measurement procedure described/reported?<br>- With respect to implementation, e.g.,<br>- The text of the questionnaire items<br>- The code/software for generating stimuli<br>- Instructions for participants<br>- Other measurement characteristics (duration of experiment, decision rules, etc.)<br>- With respect to scoring, e.g.,<br>- Sum vs. latent scores<br>- Average reaction time vs. composite of reaction time and accuracy<br>- Were factors allowed to correlate in the CFA?<br>- Was measurement invariance accounted for?<br>- Were summary statistics (i.e., means and standard deviations, not only standardised effect sizes) reported?
Measure modifications	- Did the authors report having made modifications to how each measure was previously used (or as specified by a published measurement protocol)?<br>- Were modifications transparently described and justified?

	- Was validity evidence provided for the modified procedure?<br>- Were sensitivity analyses reported?<br>- Were the possible implications of modifications and comparability to other implementations discussed?
Preregistration and Registered Reports	- Was the study pre-registered?<br>- If yes, does the pre-registration include a section on the measure(s) to be used?<br>- If yes, does it include information about nonredundancy, protocol adherence, and modifications as outlined above?<br>- If yes, were the deviations from the pre-registration transparently documented?
